# Doppler Echocardiographic Diagnosis of a Rare Pentalogy of Fallot Having Penta-Cardiac Anomalies: A Case Report

**DOI:** 10.4021/jocmr2009.09.1261

**Published:** 2009-10-16

**Authors:** Mohd Suhail, Mohd Faizul-Suhail, Hina Khan, Safia Suhail

**Affiliations:** a Department of Biochemistry, University of Allahabad, Allahabad-211002, India; b City Nursing & Maternity Home Research Center, 21, Minhajpur, Allahabad-211003, India

## Abstract

An infant, showing peripheral cyanosis, was born after lower abdominal peripheral caesarian section of the pregnant women having TORCH positive test with the infection of Toxoplasma gondiiand Cytomegalovirus. She had three abortions prior to this pregnancy. Doppler echocardiography of the baby showed profound intracardiac defects. After birth, echocardiography was carried out for diagnosis of associated cardiac anomalies. Doppler echocardiography showed pentalogy of Fallot, and the present case represents the Pentalogy of Fallot having pulmonary atresia. The baby's heart anomalies were ASD (Atrial Septal Defect - 6 mm RT to LT Shunt), VSD (Ventricular Septal Defect - bidirectional shunt), PDA (Patent Ductus Arteriosus - filling both the pulmonary arteries), and Overriding of Aorta with pulmonary atresia. In conclusion, whenever the diagnosis pentalogy of fallot is suspected, a multidisciplinary approach is essential.

**Keywords:**

Pentalogy of fallot; Overriding Aorta; Ventricular Septal Defect; Atrial Septal Defect; Pulmonary Atresia; Doppler Echocardiography

## Introduction

Tetralogy of Fallot [1], also known as Fallot's syndrome or Fallots tetrad, has four key features. A ventricular septal defect (a hole between the ventricles) and many levels of obstruction from the right ventricle to the lungs (pulmonary stenosis) are the most important. Also, the aorta (major artery from the heart to the body) lies directly over the ventricular septal defect, and the right ventricle develops thickened muscle. Because the aorta overrides the ventricular defect and there's pulmonary stenosis, blood from both ventricles (oxygen-rich and oxygen-poor) is pumped into the body. Sometimes the pulmonary valve is completely obstructed (pulmonary atresia). Infants and young children with unrepaired tetralogy of Fallot are often blue (cyanotic) as in the present case. The reason is that some oxygen-poor blood is pumped to the body.

## Case Report

**Figure 1:**
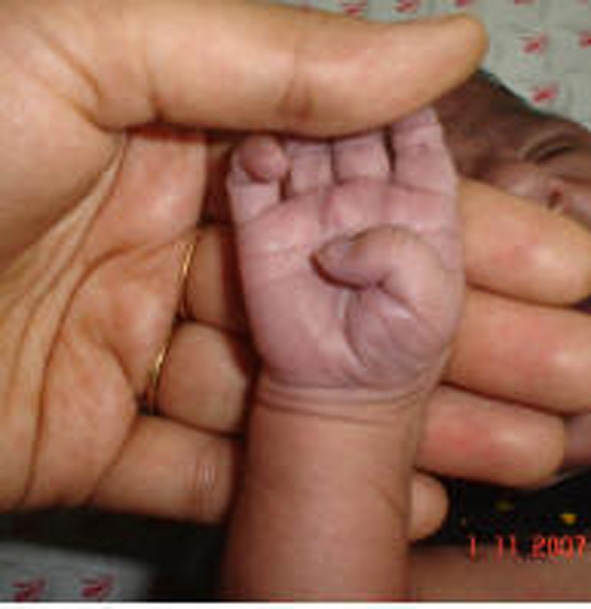
Showing peripheral cyanosis over a palm.

A 2.5 kg infant was born at 38 weeks gestation after lower abdominal caesarian section (L.A.C.S.) of 25 years old woman having breech presentation in our hospital. The female child showed peripheral cyanosis as evident from the Figure 1.

The mother having B positive blood group, was tested positive for TORCH-test. TORCH, as an acronym, stands for Toxoplasmosis, Other [T. pallidum, Varicella-zoster virus (VZV), Parvovirus B19], Rubella virus, Cytomegalovirus (CMV), and Herpes Simplex Virus (HSV). She was positive for toxoplasmosis caused by the protozoan, Toxoplasma gondiiand Cytomegalovirus (CMV). She had three abortions prior to this pregnancy. The infant is infected transplacentally after the parasites invade the placenta.The digital skiagram chest AP view of the baby showed both lung fields and CP angle to be normal, which was carried out on the third day after birth.

The baby's blood report on sixth day after birth showed Serum Bilirubin (total) to be 4.6 mg/dl, S. Bilirubin Direct 1.5 mg/dl and S. Bilirubin Indirect 3.1 mg/dl, whereas random sugar level was found to be 87.8 mg/dl which was within normal range. Blood analysis carried with Radiometer ABL77 Series showed the abnormalities regarding Hematocrit: 63% (36 - 48%); Electrolytes: K^+^ 5.5 mM/L (3.4 - 4.5 mM/L), Cl^-^ 109 mM/L (98 106 mM/L); Blood Gas at 37^o^C: pCO_2_ 54 mmHg (32 - 45 mmHg), pO_2_ 23 mmHg (83 - 108 mmHg) and pH 7.23 (7.35 - 7.45). Other detailed blood report of the baby was nearly normal. But, interestingly, the methemoglobin was found significantly high: 7.2% whereas the normal range in children < 1 year is up to 1.5% of the total hemoglobin.

In the present case, doppler echocardiography was employed, which is a procedure using ultrasound technology to examine the heart. An echocardiogram uses high frequency sound waves to create an image of the heart while the use of doppler technology allows determination of the speed and direction of blood flow by utilizing the doppler effect. Doppler echocardiography showed tetralogy of Fallot, and the present case represents the Pentalogy of Fallot with pulmonary atresia. The babys heart anomalies were ASD (Atrial Septal Defect - 6 mm RT to LT Shunt), VSD (Ventricular Septal Defect - bidirectional shunt), PDA (Patent Ductus Arteriosus - filling both the pulmonary arteries), and Overriding of Aorta with pulmonary atresia.

## Discussion

**Figure 2:**
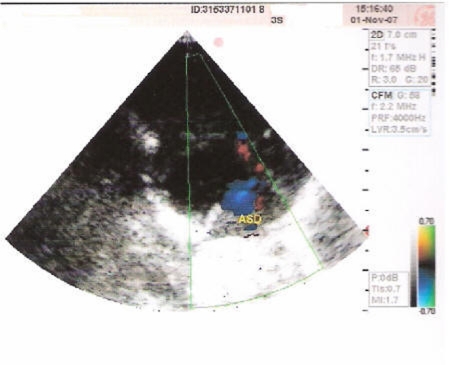
Echocardiogram showing Ostium Secundum ASD (6 mm RT TO LT SHUNT).

**Figure 3:**
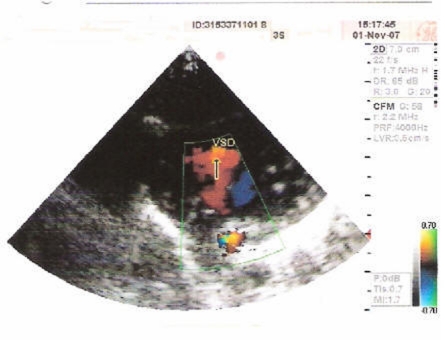
Echocardiogram showing large membranous VSD (BIDIRECTIONAL SHUNT).

**Figure 4:**
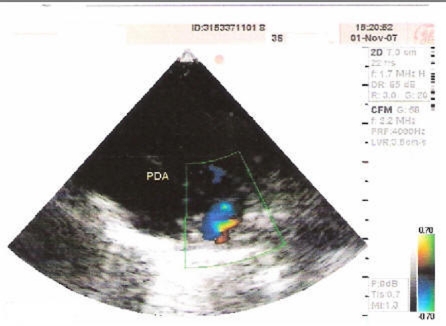
Echocardiogram showing PDA filling both the pulmonary arteries.

The first defect observed in the present case was of Atrial Septal Defect (ASD) as shown in Figure 2 is a form of congenital heart defect that enables blood flow between the left and right atria via the interatrial septum. The interatrial septum is the tissue that divides the right and left atria. Without this septum, or if there is a defect in this septum, it is possible for blood to travel from the left side of the heart to the right side of the heart, or vice versa. The second anomaly of the present case wasof Ventricular Septal Defect (VSD) as shown in Figure 3 is a defect in the ventricular septum, the wall dividing the left and right ventricles of the heart. The third defect in the present case wasof Patent Ductus Arteriosus (PDA) as evident from Figure 4 is a congenital heart defect wherein a neonate's ductus arteriosus fails to close after birth. The result is that the aorta receives some blood from the right ventricle, which reduces the amount of oxygen in the blood. Symptoms are uncommon but in the first year of life include increased work of breathing and poor weight gain. With age, the PDA may lead to congestive heart failure if left uncorrected. The fourth defect was of an overriding aorta which is a congenital heart defect where the aorta is positioned directly over a ventricular septal defect, instead of over the left ventricle. It is one of the four conditions of the Tetralogy of Fallot.

**Figure 5:**
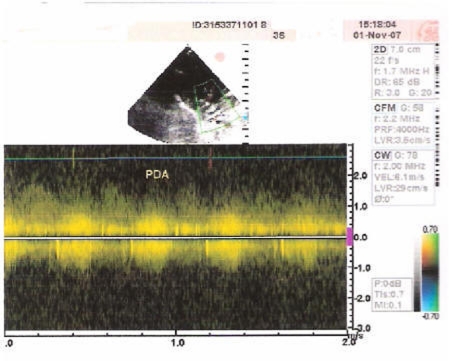
Echocardiogram showing PDA with continuous blood flow.

**Figure 6:**
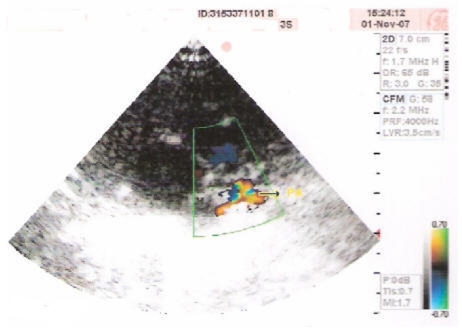
Echocardiogram showing pulmonary atresia marked with arrow.

Present case had pulmonary atresia defect also, which is a congenital malformation of the pulmonary valve in which the valve orifice fails to develop. The valve is completely closed thereby obstructing the outflow of blood from the heart to the lungs. The pulmonary valve is located on the right side of the heart between the right ventricle and pulmonary artery. In a normal functioning heart, the opening to the pulmonary valve has three flaps that open and close like one way doors. As these flaps open and close they force blood to flow forward into the pulmonary artery and backward into the right ventricle then forward again to the lungs where the blood becomes oxygenated. With the disease pulmonary atresia, the flap-like openings are completely covered by a layer of tissue, thus preventing the ability of blood flow to the lungs to become oxygenated. The body requires oxygenated blood for survival. Pulmonary atresia is not threatening to a developing fetus however, because the mother's placenta provides the needed oxygen since the baby's lungs are not yet functional. Once the baby is born, its lungs must now provide the oxygen needed for survival. But with pulmonary atresia there was no opening, as in the present case, on the pulmonary valve for blood to get to the lungs and become oxygenated, and the only source of pulmonary blood flow was a patent ductus arteriosus. In the present case, the continuous blood flow is clearly evident from Figure 5. Due to this, the newborn baby was blue in color as shown in Figure 1 and pulmonary atresia can usually be diagnosed within hours or minutes after birth. Because of the ductus arteriosus patency, the blood flow was maintained in the body and the baby could survive for few days without any cardiovascular surgery. Otherwise, due to pulmonary atresia as shown in Figure 6 baby could have not survived for such a long (12 days) duration.

The association of tetralogy of Fallot with anomalous pulmonary venous drainage is rare and only limited number of cases have been reported till now [2,3]. It is estimated that 8% of cardiac malformations are due to genetic factors, 2% to environmental agents, and the vast majority to a complex interplay between genetic and environmental influences [4]. Classic examples of environmental cardiovascular teratogens include rubella virus. In the present case, the mother giving birth to the newborn was having infection of rubella virus as well.

Mostly, the defect is due to antero-cephalad deviation of the outlet septum resulting in: (i) an unrestricted large anterior malalignment subaortic VSD; (ii) right ventricular outflow tract obstruction which may be infundibular valvular, supravalvular or a combination of all; (iii) consequent right ventricular hypertrophy; and (iv) an overriding aorta (< 50%). Accompanying features can include additional muscular VSDs, anomalous coronary arteries, a right-sided aortic arch, PDA, aortic root dilation, and aortopulmonary collaterals (mainly seen in patients with pulmonary atresia / VSD). Use of prenatal magnetic resonance imaging (MRI) may enhance the visualization of the fetal anomalies [5]. After birth, echocardiography is essential for diagnosis of associated cardiac anomalies.

Nevertheless, small defects of the diaphragm and pericardium can be extremely difficult to diagnose accurately. In these patients and in cases of possible surgical intervention, MRI might be useful [6,7]. MRI provides good delineation of the aorta, right ventricular outflow tract, VSDs, right ventricular hypertrophy, and the pulmonary artery and its branches. MRI can be used to measure intracardiac pressures, gradients, and blood flows. The treatment of the pentalogy of Fallot consists ofcorrective cardiovascular surgery. However, in the present case the mother and her attendants were advised to go for the cardiovascular surgery, but before they could decide, the child expired.

In conclusion, careful preoperative assessment is required in those with totally anomalous connections. Whenever, the diagnosis of pentalogy of Fallot is suspected, a multidisciplinary approach is essential. A prenatal medical team consisting of a gynecologist, a neonatologist, a pediatric cardiologist, a geneticist, and a pediatric surgeon should use their expertise in choosing the best approach to this severe disorder.
